# Optimal Routing in General Finite Multi-Server Queueing Networks

**DOI:** 10.1371/journal.pone.0102075

**Published:** 2014-07-10

**Authors:** Tom van Woensel, Frederico R. B. Cruz

**Affiliations:** 1 Department of Industrial Engineering & Innovation Sciences, Technische Universiteit Eindhoven, Eindhoven, The Netherlands; 2 Departamento de Estatística, Universidade Federal de Minas Gerais, Belo Horizonte, MG, Brazil; University of Nottingham, United Kingdom

## Abstract

The design of general finite multi-server queueing networks is a challenging problem that arises in many real-life situations, including computer networks, manufacturing systems, and telecommunication networks. In this paper, we examine the optimal routing problem in arbitrary configured acyclic queueing networks. The performance of the finite queueing network is evaluated with a known approximate performance evaluation method and the optimization is done by means of a heuristics based on the Powell algorithm. The proposed methodology is then applied to determine the optimal routing probability vector that maximizes the throughput of the queueing network. We show numerical results for some networks to quantify the quality of the routing vector approximations obtained.

## Introduction

The design of networks with random arrivals, random service times, multiple servers, and a finite number of buffer spaces is a challenging problem that arises in many real-life situations, *e.g.* in manufacturing systems [1], [2], computer networks [3], [4], public services [5], [6], call centers [7], [8], pedestrian and vehicular traffic [9]–[12], among other situations. This problem is challenging because finite queueing networks are notoriously difficult to analyze analytically, and closed form expressions are not easily constructed for the performance measures of such systems. Also note that the underlying network design problems involved are usually very hard to solve.

In fact, there are several distinct *network design* optimization problems associated with finite queueing networks. According to Daskalaki & Smith [13] the optimal network design problem can be split up into three interrelated optimization problems:

1. The optimal topology problem (OTOP), which deals with decisions of the design of the network itself, that is, the number of nodes (*e.g.* workstations, warehouses, delivery points, *etc.*) and arcs (*e.g.* corridors, conveyors, escalators, *etc.*) and the general configuration of these two components;
2. The optimal routing problem (OROP), which deals with determining the routing probabilities in the network defined by the first problem;
3. The optimal resource allocation problem (ORAP), which deals with the optimal allocation of the scarce resources in the network, *e.g.* the number of buffers (*i.e.*, the buffer allocation problem, BAP) and the number of servers (*i.e.*, the server allocation problem, CAP).

These three problems are challenging and difficult optimization problems. For an arbitrary topology, the OTOP is shown to be 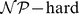 [14], and the same is conjectured for the general ORAP [15].

Previous work focused mainly on the ORAP in open finite acyclic queueing network settings. Both BAP and CAP are probably among the most well-known optimal resource allocation problems [16]. For instance, Cruz *et al.* [17] and Smith *et al.* [18] looked into the BAP, both in a single and in a multi-server setting, and Smith *et al.* [19] proposed algorithms to solve the CAP. However, the routing probabilities are usually assumed to be known beforehand for BAP and CAP [20].

The overall research objective of this paper is to build a relevant model and solution methodology for the system's throughput maximization problem. In this paper, we focus on optimizing the routing probabilities through the queueing network, i.e. the OROP. A similar research question is tackled by Daskalaki & Smith [13] in which they evaluated the joint effect of buffer allocation and routing on the throughput. Earlier, Gosavi & Smith [21] focused on the routing optimization problem related to the overall objective of throughput maximization. The common ground of both papers is that they used queueing networks with *single* servers and *exponential* service times [13], [21]. Kerbache & Smith [22] considered, for different product classes, the optimal routes conjoint with a variant of the optimal topology problem to determine the connected arcs in a *single* server queueing network setting. Distinct from Daskalaki & Smith [13] and Gosavi & Smith [21] is that Kerbache & Smith [22] considered general arrival times, general service times, and single server queues. Secondly, Gosavi & Smith [21] did not consider the general expansion method (GEM) in their analysis as the evaluation tool [23].

Specifically, we examine the OROP, by means of a combination of the GEM and a heuristic based on the Powell algorithm [24], specifically for acyclic networks of 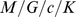 queues, which in Kendall notation means a queueing system with **M**arkovian arrival rates, **G**eneral service times, **c** parallel servers, and a total capacity of **K** items, *including* those items in service (practical applications to 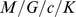 queueing networks include manufacturing and service systems [25] and transportation and material handling systems [26]). The results are compared to simulations to attest for the quality of the routing vectors obtained. Besides, another important contribution of this paper is to present helpful approximations to swift managerial decisions regarding the optimal routing probability vectors to maximize the overall throughput in a network of finite general-service queues. We also present important empirical arguments to show that these approximations are effective.

This paper is organized as follows. The next section describes in detail the mathematical model formulation considered and elaborates on both the performance evaluation tool and on the optimization procedure. In the following section detailed results are given for the problem on hand. Finally, the last section concludes this paper with final remarks and topics for future work in the area.

## Materials and Methods

### Mathematical Programing Formulation

The problem is defined on a digraph 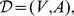 in which 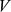 is the set of vertexes (finite queues) and 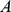 is the set of arc (connections between the queues). Each vertex (queue) is characterized by Poisson arrivals, general service, and multiple servers. Mathematically, the optimal routing problem can be formulated as follows.

(OROP):
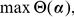
(1)subject to:
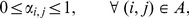
(2)

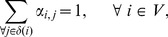
(3)in which 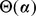 is the throughput, which is the objective that must be maximized, 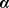 the optimal routing probability matrix, 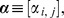 *i.e.* the matrix that maximizes the objective function of this predefined network, and 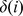 is the set of succeeding vertexes of vertex 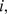 that is, 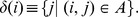


The throughput will thus be affected by the effective routing of jobs through the network, the variability of the service distribution, the number of servers, and the number of buffers. It should be clear that the above model is a highly difficult stochastic programming problem to handle due to the inherent non-linearity of the objective function combined with the absence of any closed-form expressions for the throughput 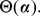


### Proposed Algorithm

**Figure 1:**
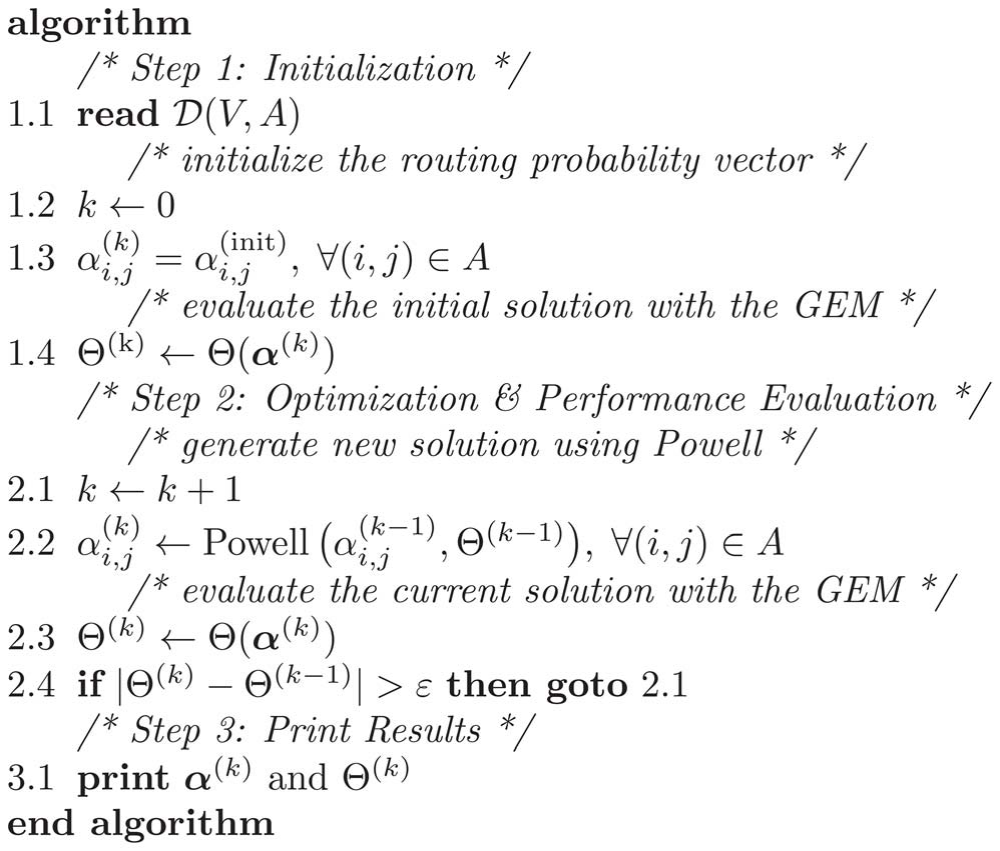
Structured overview of the methodology.

Figure 1 presents the proposed algorithm to solve the OROP in order to provide more insights into the interaction between the performance evaluation tool and the optimization method.

The initial routing probability vector is conveniently set to the inverse of the number of nodes after a split,
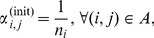
(4)in which 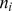 is the number of succeeding nodes to node 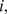 that is, the cardinality of set 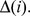 The optimization step itself is an iteration in which new solutions are generated following the Powell logic until convergence, that is, until the difference in 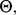 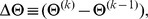 is less than a predefined tolerance 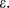


The Powell algorithm can be described as an unconstrained optimization procedure that does not require the calculation of first derivatives of the function. Numerical examples have shown that the method is capable of minimizing a function with up to twenty variables [24], [27]. The Powell method locates the minimum of a non-linear function 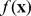 by successive uni-dimensional searches from an initial starting point 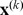 along a set of conjugate directions. These conjugate directions are generated within the procedure itself. The Powell method is based on the idea that if a minimum of a non-linear function 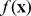 is found along 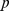 conjugate directions in a stage of the search, and an appropriate step is made in each direction, the overall step from the beginning to the *p*-th step is conjugate to all of the 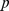 sub-directions of the search. We have seen remarkable success in the past with coupling Powell algorithm and the GEM [19]. We discuss the GEM in detail now, which is also the method we used to obtain the performance measures for the problem studied in this paper.

### Performance Evaluation

In previous papers (see *e.g.* Kerbache & Smith [23], [28]) the GEM has been successfully used to evaluate the performance measures of acyclic networks of finite queues. The GEM is a robust and effective approximation technique that is basically a combination of repeated trials and node-by-node decomposition in which each queue is analyzed separately and then corrections are made in order to take into account the interrelation between the queues in the network. The GEM has three stages, *Network Reconfiguration*, *Parameter Estimation*, and *Feedback Elimination*, to be described as follows.

#### Stage I: Network Reconfiguration

**Figure 2:**
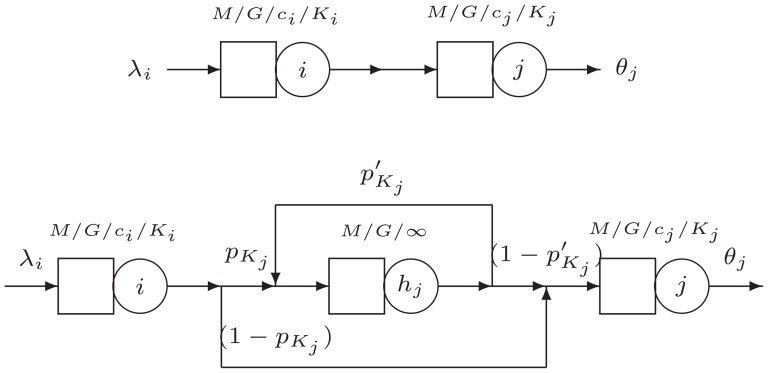
The generalized expansion method.

The first step in the GEM involves reconfiguring the network. An artificial vertex 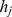 is added preceding each finite vertex 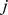 in the network. The artificial vertex is added to register the blocked customers at node 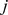 and is modeled as an 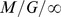 queue, as shown in Figure 2.

When an entity leaves vertex 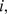 vertex 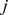 may be blocked with probability 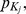 or unblocked, with probability 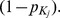 Under blocking, the entities are rerouted to vertex 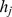 for a delay while node 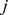 is busy. Vertex 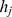 helps to accumulate the time an entity has to wait before entering vertex 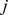 and to compute the effective arrival rate to vertex 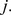 In other words, the GEM ultimate goal is to provide an approximation scheme to *update* the service rates at the upstream vertex 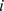 to take into account all blocking after service caused by the downstream vertex 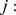


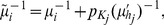
(5)in which 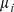 is the exponential service rate at vertex 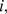 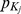 is the blocking probability of finite queue 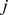 of size 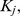 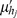 is the corrected exponential service rate at the artificial vertex 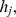 and 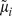 is the *updated* service rate at vertex 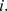 As a final note, an important point about this process is that we do not physically modify the networks, only represent the expansion process through the software.

#### Stage II: Parameter Estimation

In the second stage, the parameters 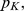 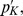 and 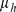 must be estimated, which is done essentially by utilizing known results for queueing theory. Index 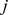 is omitted for simplicity.


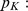
 Analytical results from the 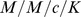 queue provide the following expression for the blocking probability 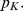


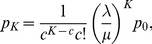
(6)in which
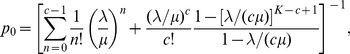
(7)for 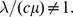


However, the interest is on 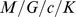 models, for which there is not exact closed form expression for 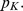 Then approximations must be used and Kimura's [29] two moment approximation has proven to be very effective in similar cases [25], [30]. For example, let us fix 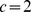 and the following closed form expression can be developed from Equation (6), for the optimal buffer size 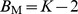 for Markovian 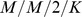 queues, as a function of the blocking probability:
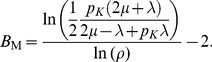
(8)


The following Kimura's two moment approximation can be used to approximate the optimal buffer size 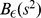 of a general service 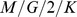 queue:
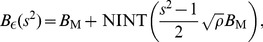
(9)in which 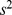 is the squared coefficient of variation of the service time distribution at the queue, 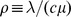 is the traffic intensity, 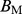 is the exact buffer size for a respective Markovian system, and 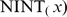 is the nearest integer to 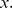 Now, if we invert Equation (9) to solve for 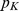 we achieve:
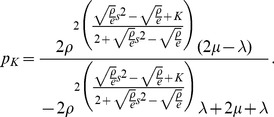
(10)


This is a process that can be extended for 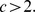 In fact, expressions for 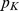 of up to 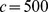 are available [30]. Another expressions, for 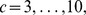 are included in the software so that we have a complete set of blocking probabilities for 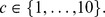



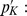
 Since there is no closed form solution for this quantity the following approximation is used given by Labetoulle & Pujolle [31] obtained using diffusion techniques.
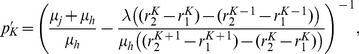
(11)in which 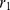 and 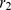 are the roots to the polynomial
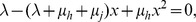
with 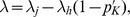 in which 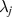 and 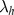 are the actual arrival rates to the finite and artificial holding nodes respectively. Furthermore, the arrival rate to the finite node 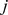 is given by:
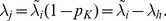




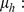
 The delay distribution of a blocked customer at the holding node has the same distribution as the remaining service time of the customer being serviced at the node doing the blocking. Using renewal theory, one can show that the remaining service time distribution has the following rate 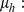


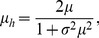
(12)in which 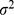 is the service time variance given by Kleinrock [32]. Notice that if the service time distribution at the finite queue doing the blocking is exponential with rate 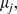 then:
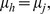
that is, the service time at the artificial node is also exponentially distributed with rate 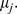 When the service time of the blocking node is not exponential, then 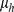 will be affected by 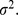


#### Stage III: Feedback Elimination

This stage is simply to eliminate the feedback loop, by recomputing the service time at vertex 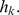 The updated service rate is given by:
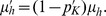



#### Summary

Similar equations can be established with respect to each of the finite vertexes (queues). Ultimately, we have simultaneous non-linear equations in variables 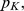 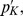 and 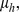 along with auxiliary variables such as 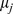 and 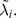 Solving these equations simultaneously, we can compute all performance measures of the network.

## Numerical Results and Discussion

The software implementation is currently in Fortran 77. The compiler used was the DIGITAL Visual Fortran, Version 6, with the IMSL Fortran 90 MP Library version 3.0 for Microsoft Windows, to solve the nonlinear equations from the GEM. In our implementation, we set 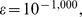 which proved to be effective based on the experiments. We first discuss the shape of the objective function. Secondly, we will give more insights for a number of split structures. We end the numerical results with some complex network structures. Please bear in mind that the range of possible experiments is exponential itself, so we have determined a very selected, but representative sample to present.

### Shape of the Objective Function

**Figure 3:**
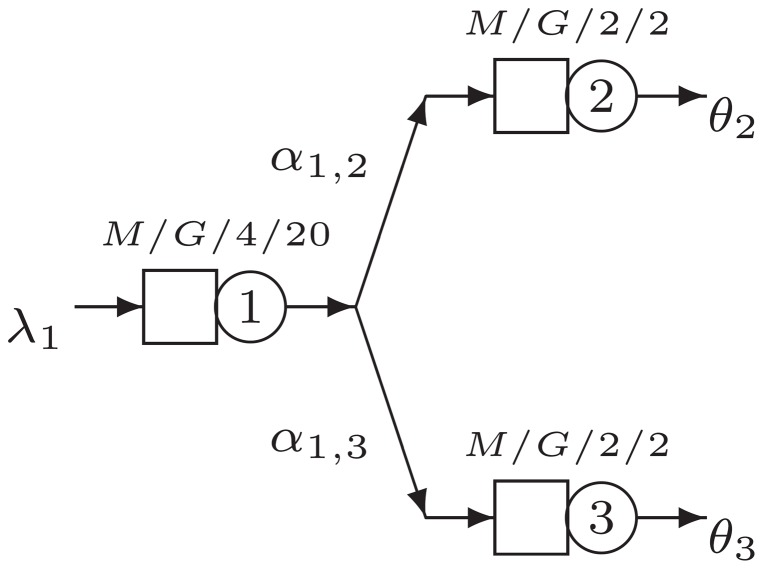
Basic split network B1.

It is interesting to analyze the shape of the objective function for the optimization problem described earlier. The case discussed here is defined as follows. We have a three node network with a split into two branches, as seen in Figure 3. The general parameters for the base case are 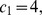 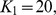 and 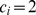 and 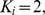 for 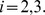 The criteria to select those parameters is such that the number of servers 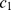 and the total capacity 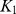 of node 1 is larger than the others as to prevent it from becoming a bottleneck.

**Figure 4:**
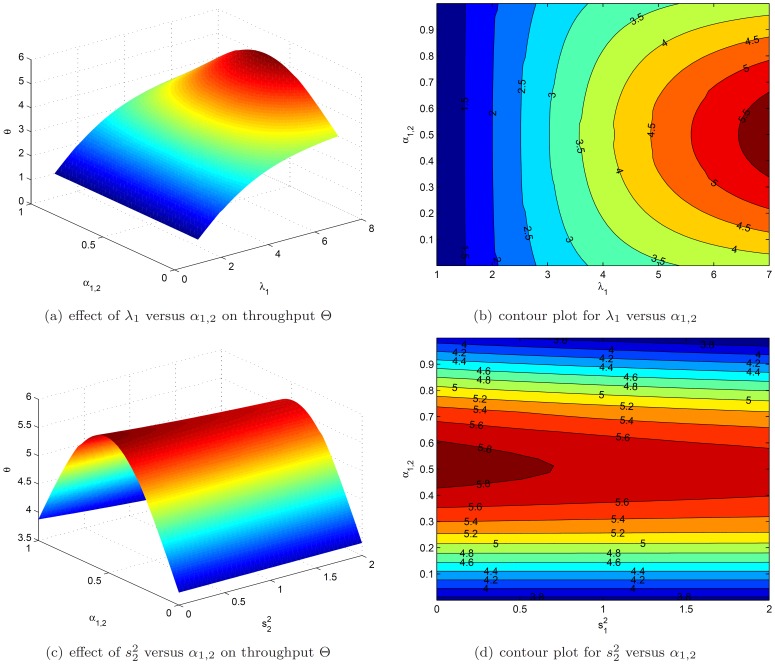
The shape of the objective function.

We are particularly interested in the relationship between the overall throughput 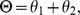 the routing probability 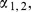 the arrival rate 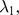 and the squared coefficient of variation of node 2, 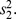 Consequently, we set 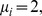 for all nodes, and 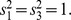 The sensitivity of these settings on the throughput is not analyzed now, but is amongst others the subject of study in the next sections. Next, we enumerate all possible combinations for 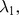 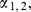 and 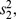 and then analytically obtain the corresponding throughput 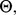 which is shown in Figure 4 (always on the vertical axis), as a function of 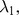 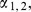 and 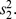


Figure 4-(a) clearly shows that the arrival rate is interacting with the routing probability. For low arrival rates, the network has low utilization. Consequently, different routing probabilities do not result in large changes in throughput 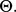 On the other hand, for large arrival rates, 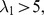 one clearly sees an optimal point in regard to the routing probability. Due to the symmetrical structure considered, a 50% split seems to be optimal here. Figure 4-(b) looks into the joint effect of changing the squared coefficient of variation, 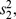 together with the routing probability 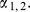 Again the inverted U-shape effect with a maximum around the 50% routing probability is visible. Next to this, it is clear that increasing the squared coefficient of variation from 0 to 2 reduces the overall throughput 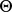 but has a smaller impact on throughput than the routing probability. Based on this simple network structure, it is evident that the routing probabilities and the squared coefficient of variation affect the throughput to a large extent. Consequently, correctly setting the routing matrix 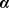 leads to significant gains in terms of throughput.

### Basic Split Networks

**Figure 5:**
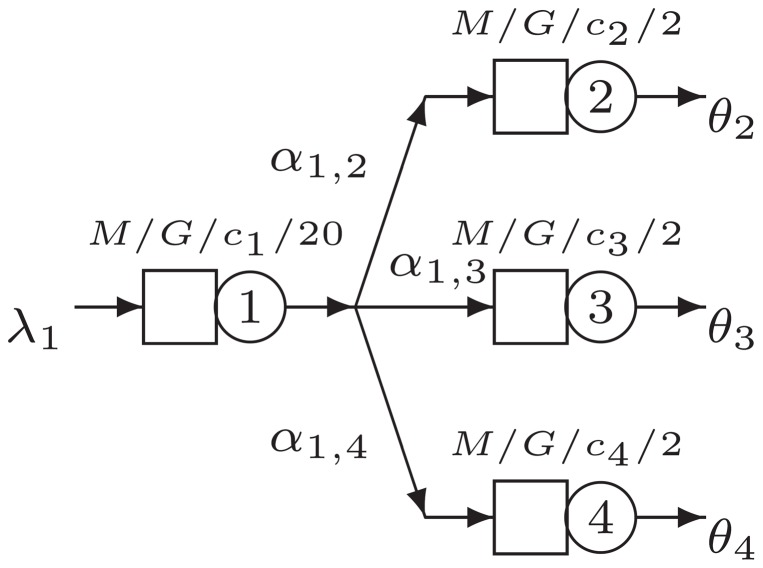
Basic split network B2.

In this section, we analyze further the two-branch network from Figure 3 and include in our analysis the three-branch network seen in Figure 5. We are interested in assessing the influence of the number of servers 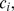 total capacities 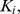 service rates 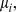 and squared coefficient of variation of the service times 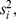 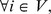 in the model OROP, Equations (1) – (3). We choose to start with these two variants of a basic split structure as, from a routing allocation point of view, splits are the key building blocks in a generally configured network. The nodes after the splits are the ones of interest here. The first buffer 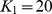 is larger than the others 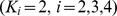 as this will help to prevent the first queue of becoming a bottleneck node. The arrival rate 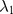 is set equal to values 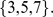


#### Split with Two Branches

The top part of Table 1 gives the results for a two-branch split networks for unbalanced service rates 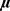 and different squared coefficients of variation 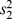 and 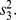 In these cases the service rate of node 2 is made either relatively lower 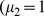 versus 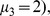 or equal 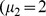 versus 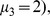 or higher 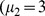 versus 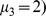 than the service rate of node 3. The base cases (sets B1d to B1f) show that the routing probability is roughly equal to 0.5 when the nodes after the split are identical (that is, same number of servers, capacities, service rates, and squared coefficient of variation). Moreover, this results appears to be insensitive to changes in the squared coefficient of variation of both nodes after the split. Of course, the throughput 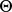 is affected (reduced) due to the changes (increase) in the variability. Secondly, changing the service rate of node 2, 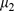 (and keeping all other parameters equal to the base case settings), clearly shows that the fast nodes receive preference over the slow nodes. For example, in sets B1a to B1c (*i.e.*, when node 2 is slower than node 3) a lower routing probability is set to node 2 (0.3334) than the one to node 3 (0.6666).

**Table 1:** Results for two-branch split networks.

			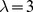			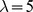			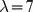		
Set	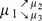	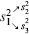	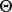	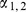	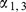	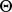	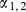	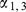	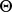	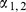	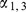
B1a	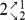	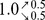	2.9165	0.3337	0.6663	4.2950	0.3334	0.6666	5.0930	0.3334	0.6666
B1b		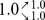	2.9004	0.3334	0.6666	4.2193	0.3334	0.6666	4.9819	0.3334	0.6666
B1c		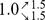	2.8850	0.3337	0.6663	4.2950	0.3334	0.6666	4.8888	0.3334	0.6666
B1d	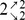	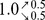	2.9733	0.4989	0.5011	4.6545	0.5000	0.5000	5.8777	0.5000	0.5000
B1e		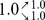	2.9672	0.4986	0.5014	4.6010	0.5000	0.5000	5.7694	0.5000	0.5000
B1f		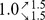	2.9609	0.4984	0.5016	4.5525	0.5000	0.5000	5.6765	0.5000	0.5000
B1g	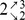	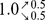	2.9878	0.5993	0.4007	4.8807	0.6000	0.4000	6.3176	0.6000	0.4000
B1h		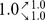	2.9847	0.5993	0.4007	4.8568	0.6000	0.4000	6.2305	0.6001	0.3999
B1i		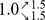	2.9816	0.5990	0.4010	4.8337	0.6000	0.4000	6.1529	0.5998	0.4002
B1j	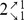	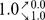	2.6743	0.5222	0.4778	3.5007	0.5039	0.4961	3.7981	0.4843	0.5167
B1k		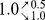	2.6489	0.5100	0.4900	3.4544	0.5021	0.4979	3.7573	0.4944	0.5056
B1l		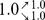	2.6278	0.5000	0.5000	3.4161	0.5000	0.5000	3.7212	0.5000	0.5000
B1m		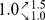	2.6101	0.4918	0.5082	3.3839	0.4978	0.5022	3.6898	0.5035	0.4965
B1n		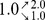	2.5947	0.4850	0.5150	3.3567	0.4960	0.5040	3.6626	0.5059	0.4941
B1o	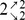	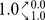	2.9739	0.5309	0.4690	4.6612	0.5265	0.4735	5.8910	0.5181	0.4819
B1p		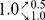	2.9704	0.5132	0.4868	4.6286	0.5116	0.4884	5.8242	0.5081	0.4919
B1q		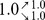	2.9671	0.4986	0.5014	4.6010	0.4999	0.5001	5.7694	0.5000	0.5000
B1r		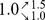	2.9642	0.4864	0.5136	4.5773	0.4903	0.5097	5.7234	0.4933	0.5067
B1s		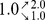	2.9615	0.4758	0.5242	4.5567	0.4823	0.5177	5.6842	0.4877	0.5123
B1t	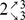	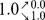	2.9938	0.5341	0.4659	4.9372	0.5283	0.4717	6.6047	0.5690	0.4310
B1u		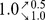	2.9929	0.5133	0.4847	4.9289	0.5107	0.4893	6.5319	0.5125	0.4875
B1v		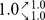	2.9920	0.4995	0.5005	4.9214	0.4965	0.5035	6.5039	0.4314	0.5686
B1w		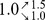	2.9912	0.4857	0.5143	4.9147	0.4844	0.5156	6.4891	0.4314	0.5686
B1x		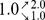	2.9905	0.4742	0.5258	4.9086	0.4741	0.5259	6.4749	0.4314	0.5686

Rather than changing the squared coefficient of variation of both nodes after the split, we evaluated some unbalanced cases where only node 2 is affected by a different squared coefficient of variation, 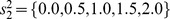 (sets B1j to B1x, Table 1), combined with 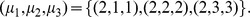 For these cases, we observe that the unbalance caused by the squared coefficient of variation only slightly changes the routing probability compared to sets with equal squared coefficients of variation (sets B1l, B1q, and B1v, Table 1). This is a confirmation of what we observed when evaluating the objective function earlier in the previous section. As we are now focusing on the small scale networks, this conclusion does not mean that the squared coefficient of variation has little effect *in general*. It is interesting to see that the throughput value is reducing as the squared coefficient of variation goes up although the routing probability is changing to protect more against the uncertainty in the second node. This is more prevalent in highly loaded systems.

For the two-branch split networks, we evaluated a number of routing vectors around the optimal routing obtained. Table 2 shows that the algorithm seems to have reached the optimal allocation for the routing probabilities into nodes 2 and 3 (set B1e, Table 1). Of course, one might argue that the optimization is rather easy due to the symmetric setting of the parameters. Therefore, we did the same analysis for the same parameter settings but with a network with unbalance in the service rates (set B1b, Table 1), also seen in Table 2.

**Table 2:** Perturbations around the optimal solution of two-branch split networks.

		Set B1e (balanced)			Set B1b (unbalanced)		
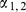	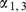	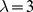	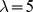	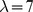	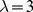	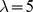	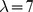
0.10	0.90	2.7388	3.7815	4.3370	2.7386	3.7803	4.3314
0.20	0.80	2.8275	4.1084	4.9123	2.8247	4.0821	4.7797
0.30	0.70	2.8935	4.3793	5.3672	2.8769	4.2099	4.9706
**0.33**	**0.66**	…	…	…	**2.9004**	**4.2193**	**4.9819**
0.40	0.60	2.9582	4.5697	5.6665	2.8777	4.1834	4.9412
0.45	0.55	2.9649	4.5858	5.7435	…	…	…
**0.50**	**0.50**	**2.9672**	**4.6010**	**5.7693**	2.7974	4.0086	4.7452
0.55	0.45	2.9647	4.5858	5.7435	…	…	…
0.60	0.40	2.9582	4.5697	5.6665	2.6653	3.7442	4.4038
0.70	0.30	2.8935	4.3794	5.3672	2.4904	3.3459	3.9249
0.80	0.20	2.8275	4.1084	4.9124	2.2828	2.8989	3.3374
0.90	0.10	2.7388	3.7815	4.3370	2.0514	2.4274	3.1235

In conclusion, we observed that in previous results the optimization algorithm tries to balance out the flow taking into account the differences (in service rates and squared coefficient of variation) among the two nodes after the split, which is intuitively logical as this strategy leads to the highest throughput. Moreover, the methodology seems to always find the optimal routing vector.

#### Split with Three Branches

Let us now turn to the three-branch split network, Figure 5. It would be interesting to see to what extent the optimization algorithm balances the flow over the three nodes after the split and to what extent this is affected by the characteristics of the different nodes after the split. Table 3 shows a selected set of experiments done for this specific case.

**Table 3:** Results for three-branch split networks.

			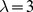				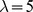				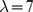			
Set	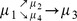	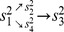	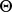	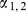	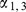	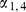	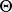	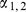	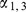	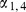	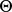	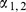	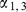	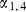
B2a	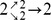	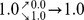	2.9933	0.3758	0.3120	0.3122	4.9326	0.3587	0.3204	0.3209	6.5642	0.3845	0.3333	0.2822
B2b		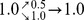	2.9926	0.3469	0.3265	0.3265	4.9268	0.3431	0.3283	0.3287	6.5209	0.3445	0.3278	0.3277
B2c		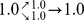	2.9921	0.3330	0.3335	0.3335	4.9217	0.3304	0.3346	0.3350	6.4982	0.3333	0.3333	0.3333
B2d		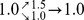	2.9915	0.3210	0.3395	0.3395	4.9174	0.3199	0.3399	0.3401	6.4973	0.2866	0.3580	0.3554
B2e		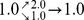	2.9910	0.3109	0.3445	0.3446	4.9135	0.3109	0.3444	0.3446	6.4623	0.3165	0.3417	0.3418
B2f	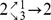	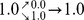	2.9927	0.1860	0.3257	0.4883	4.9277	0.1821	0.3267	0.4912	6.5239	0.1824	0.3270	0.4906
B2g		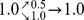	2.9923	0.1742	0.3308	0.4949	4.9246	0.1717	0.3310	0.4973	6.5097	0.1737	0.3305	0.4957
B2h		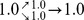	2.9921	0.1655	0.3340	0.5005	4.9221	0.1642	0.3340	0.5018	6.5034	0.2054	0.3634	0.4311
B2i		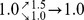	2.9918	0.1582	0.3368	0.5050	4.9199	0.1581	0.3365	0.5053	6.4886	0.1609	0.3356	0.5035
B2j		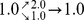	2.9916	0.1522	0.3391	0.5087	4.9181	0.1523	0.3389	0.5088	6.4806	0.1562	0.3375	0.5063

Table 3 shows that for the complete symmetric case, that is, set B2c, Table 3, again the routing probabilities are symmetric, *i.e.* 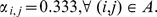 For the unbalanced cases in the squared coefficient of variation (sets B2a, B2b, B2d, and B2e, Table 3), it can be observed that the routing probability into the two identical nodes 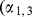 and 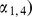 are close to each other. For the remaining asymmetrical cases (sets B2f to B2o, Table 3), again the same conclusion holds. The faster (either in high number of servers or service rates) or more reliable (in terms of low squared coefficient of variation) are the nodes, more favored they are, resulting in high routing probabilities into these nodes.

### Complex Networks

**Figure 6:**
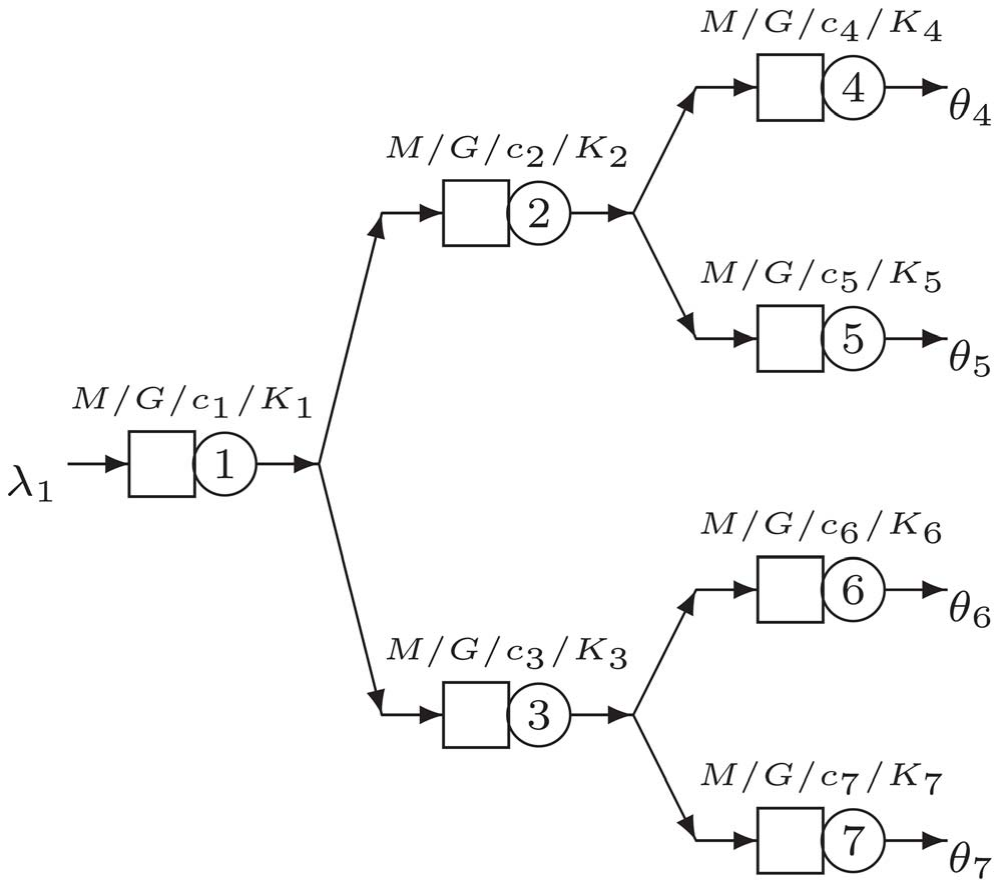
Network structure C1.

The simple networks discussed so far are interesting as they make it possible to show the behavior and logic of the optimization model in the presence of one split. In this section, we will evaluate some different complex topologies with regard to their routing probabilities. The first complex network considered is an extension of the two- and three-branch split networks, as depicted in Figure 6.

Table 4 gives an overview of a selected set of experiments for the structure C1. The initial setting is again a balanced case, that is, 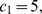 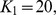 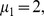 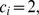 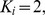 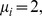 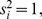 for 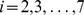 (set C1a, Table 4). Additional set of experiments involves unbalancing the service rates 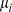, the squared coefficients of variation 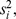 and the number of servers 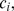 for nodes 6 and 7. With these experiments, we evaluate whether and how the methodology takes the characteristics of the complete sub-network after the split into account in determining the optimal routing vector.

**Table 4:** Results for network structure C1.

		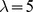							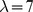						
	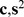	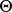	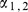	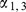	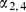	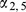	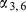	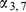	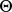	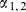	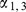	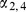	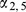	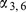	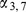
C1a	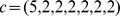														
	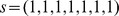	4.5810	0.5000	0.5000	0.4995	0.5005	0.5000	0.5000	5.7142	0.5000	0.5000	0.4986	0.5014	0.4999	0.5001
C1b	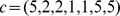														
	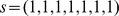	4.4978	0.4000	0.6000	0.4924	0.5076	0.5000	0.5000	6.0645	0.4484	0.5516	0.4833	0.5167	0.5000	0.5000
C1c	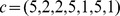														
	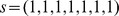	4.6002	0.5000	0.5000	0.9251	0.0749	0.9239	0.0761	6.4139	0.5000	0.5000	0.9013	0.0987	0.9013	0.0987
C1d	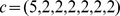														
	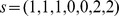	4.5808	0.5042	0.4958	0.4992	0.5008	0.4999	0.5001	6.3335	0.5047	0.4953	0.4993	0.5007	0.4996	0.5004
C1e	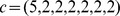														
	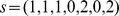	4.5826	0.5000	0.5000	0.5577	0.4423	0.5581	0.4419	6.3395	0.4999	0.5001	0.4452	0.5548	0.4464	0.5536

We set up the experiments in such a way that either there are slow nodes (experiments C1c, C1e, C1g and C1h) or slow subsystems consisting of three connected nodes (experiments C1b, C1d, C1f, and C1i). Based on Table 4, we observe that in general the slower part of the network tends to receive less flow due to a lower routing probability into the relevant part. When after the first split in node 1 there is the choice to go to either the fast or slow subsystem, the faster subsystem is preferred. This is very clear in experiments C1b, C1d, C1f, and C1i, when the routing probability always favors the fastest downstream subsystem. However, if the last nodes are different (experiments C1c, C1e, C1g, and C1h), the conclusion is different. In all these experiments, the first split is just exactly half. The imbalance in the last nodes (*i.e.* nodes 4, 5, 6, and 7 are different), is completely absorbed in the routing probability at the immediately preceding nodes (i.e. nodes 2 and 3). Interestingly, this effect did not propagate upstream and did not affect the routing at the first split. Again, we see that the effect of the squared coefficient of variation on the routing probability is smaller compared to the number of servers or the service rates.

**Figure 7:**
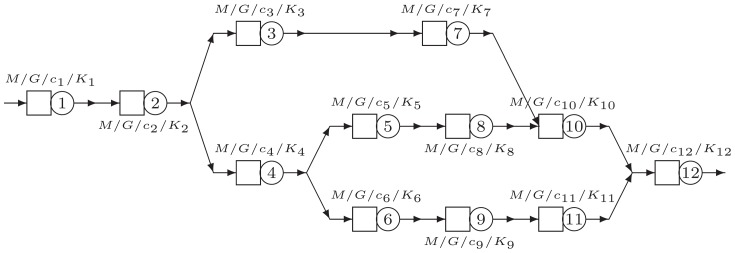
Network structure C2.

The second network structure C2 has a more general structure than the other networks, as seen in Figure 7. Nodes 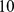 and 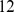 can act as a bottleneck node which might become overloaded depending upon the specific parameters. It is then interesting to see how the routing probabilities are adapted to avoid or to reduce the workload in these bottleneck nodes.

Table 5 gives the results for a selection of parameter settings for network structure C2. The table shows that when node 10 becomes the bottleneck, routing the jobs into the direction of node 10 is avoided by reducing the routing probability at node 3 (always around 0.4) and node 5 (ranging between 0.0409 to 0.4751). On the other hand, when the characteristics of node 10 are such that it is not a bottleneck, then the routing to nodes 5 and 6 is almost 50/50. Secondly, it is clear that if the last node (node 12) becomes the bottleneck, only the throughput will be reduced.

**Table 5:** Results for network structure C2.

		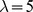					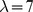				
		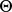	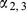	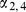	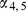	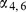	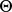	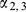	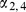	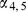	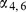
C2a	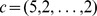										
	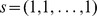	3.1719	0.4000	0.6000	0.2404	0.7596	3.3502	0.4464	0.5536	0.3500	0.6500
C2b	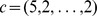										
	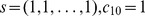	2.9699	0.4000	0.6000	0.0409	0.9591	3.0735	0.2857	0.7143	0.0256	0.9744
C2c	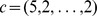										
	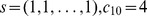	3.2660	0.4000	0.6000	0.4858	0.5142	3.4898	0.4476	0.5524	0.4679	0.5321
C2d	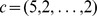										
	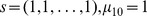	2.9251	0.3988	0.6012	0.2404	0.7596	3.0850	0.4292	0.5708	0.0002	0.9998
C2e	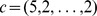										
	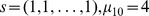	3.2611	0.3987	0.6013	0.4751	0.5249	3.4854	0.4473	0.5527	0.4637	0.5363
C2f	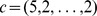										
	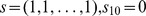	3.1929	0.4000	0.6000	0.2748	0.7252	3.3820	0.4456	0.5544	0.3473	0.6527
C2g	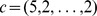										
	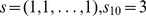	3.1407	0.4000	0.6000	0.2415	0.7585	3.3075	0.4444	0.5556	0.3149	0.6851
C2h	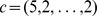										
	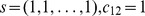	1.8784	0.3997	0.6003	0.2405	0.7595	1.9103	0.4464	0.5536	0.3500	0.6500
C2i	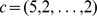										
	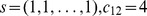	4.0567	0.4000	0.6000	0.2397	0.7603	4.5422	0.4418	0.5582	0.3500	0.6500
C2j	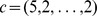										
	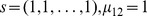	1.9113	0.3999	0.6001	0.2391	0.7609	1.9368	0.4464	0.5536	0.3500	0.6500
C2k	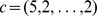										
	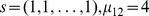	3.8888	0.4240	0.5760	0.4871	0.5129	4.4126	0.4418	0.5582	0.3500	0.6500
C2l	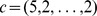										
	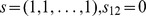	3.3224	0.4000	0.6000	0.2411	0.7589	3.5158	0.4464	0.5536	0.3500	0.6500
C2m	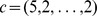										
	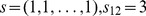	2.9862	0.4000	0.6000	0.2394	0.7606	3.1482	0.4464	0.5536	0.3500	0.6500

### Approximations for the Routing Probabilities

From a managerial point of view, it is interesting to have some good easy approximations that can be used to quickly set the routing probabilities. A number of possible approximations for the routing probabilities in the arc 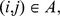 after a split of vertex 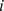 into 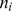 vertexes, can be considered.
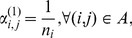
(13)

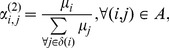
(14)

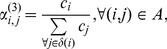
(15)

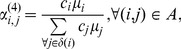
(16)in which 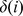 is the set of succeeding vertexes of vertex 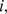 that is, 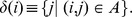 Notice that 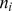 is the cardinality of set 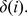


The first approximation, Equation (13), is simple but does not use any information from the 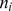 vertexes after the split. This approximation only provides an equal spread of the throughput over the succeeding vertexes. It is expected that this approximation works well when the nodes after the split are very similar in terms of service rate, number of servers, and so on. The other approximations, Equations (14), (15), and (16), do take more information into account. Equation (16) is believed to be the most general as it combines information in regard to the speed and the number of servers. On the other hand, no information about the squared coefficient of variation is included in none of the approximations.

Tables 6 and 7 show that the performance of approximation 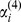 improves as the network becomes more unbalanced (for instance, cases B1a-B1c are unbalanced, as defined in Table 1, and for these cases the smallest 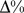 found is for 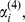 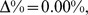 on the other hand cases B1d-B1f are balanced and for them all 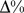 is equal to 0.00%). This approximation of course takes into account the most information from the nodes after the split (not taking into account the squared coefficient of variation). If the nodes after the split are more alike (balanced) then the second approximation becomes favorable. On the other hand the first approximation 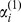 is performing acceptable as well and could be preferred due to the easy implementation.

**Table 6:** Evaluating the approximations for some B-sets.

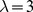		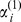		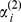		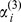		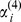	
Set	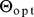	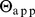	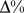	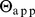	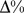	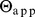	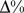	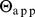	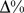
B1a	2.9165	2.8211	3.27%	2.9165	0.00%	2.8211	3.27%	2.9165	0.00%
B1b	2.9004	2.7974	3.55%	2.9004	0.00%	2.7974	3.55%	2.9004	0.00%
B1c	2.8850	2.7762	3.77%	2.8850	0.00%	2.7762	3.77%	2.8850	0.00%
B1d	2.9733	2.9733	0.00%	2.9733	0.00%	2.9733	0.00%	2.9733	0.00%
B1e	2.9672	2.9672	0.00%	2.9672	0.00%	2.9672	0.00%	2.9672	0.00%
B1f	2.9609	2.9609	0.00%	2.9609	0.00%	2.9609	0.00%	2.9609	0.00%
B1g	2.9878	2.9835	0.14%	2.9878	0.00%	2.9835	0.14%	2.9878	0.00%
B1h	2.9847	2.9797	0.17%	2.9847	0.00%	2.9797	0.17%	2.9847	0.00%
B1i	2.9816	2.9758	0.19%	2.9816	0.00%	2.9758	0.19%	2.9816	0.00%
Avg			1.23%		0.00%		1.23%		0.00%
Min			0.00%		0.00%		0.00%		0.00%
Max			3.77%		0.00%		3.77%		0.00%
B1j	2.6743	2.6721	0.08%	2.6721	0.08%	2.6721	0.08%	2.6721	0.08%
B1k	2.6489	2.6485	0.02%	2.6485	0.02%	2.6485	0.02%	2.6485	0.02%
B1l	2.6278	2.6278	0.00%	2.6278	0.00%	2.6278	0.00%	2.6278	0.00%
B1m	2.6101	2.6098	0.01%	2.6098	0.01%	2.6098	0.01%	2.6098	0.01%
B1n	2.5947	2.5938	0.03%	2.5938	0.03%	2.5938	0.03%	2.5938	0.03%
B1o	2.9739	2.9732	0.02%	2.9732	0.02%	2.9732	0.02%	2.9732	0.02%
B1p	2.9704	2.9702	0.01%	2.9702	0.01%	2.9702	0.01%	2.9702	0.01%
B1q	2.9671	2.9671	0.00%	2.9671	0.00%	2.9671	0.00%	2.9671	0.00%
B1r	2.9642	2.9640	0.01%	2.9640	0.01%	2.9640	0.01%	2.9640	0.01%
B1s	2.9615	2.9609	0.02%	2.9609	0.02%	2.9609	0.02%	2.9609	0.02%
B1t	2.9938	2.9936	0.01%	2.9936	0.01%	2.9936	0.01%	2.9936	0.01%
B1u	2.9929	2.9928	0.00%	2.9928	0.00%	2.9928	0.00%	2.9928	0.00%
B1v	2.9920	2.9920	0.00%	2.9920	0.00%	2.9920	0.00%	2.9920	0.00%
B1w	2.9912	2.9912	0.00%	2.9912	0.00%	2.9912	0.00%	2.9912	0.00%
B1x	2.9905	2.9903	0.01%	2.9903	0.01%	2.9903	0.01%	2.9903	0.01%
Avg			0.01%		0.01%		0.01%		0.01%
Min			0.00%		0.00%		0.00%		0.00%
Max			0.08%		0.08%		0.08%		0.08%
B2a	2.9933	2.9931	0.01%	2.9931	0.01%	2.9931	0.01%	2.9931	0.01%
B2b	2.9926	2.9926	0.00%	2.9926	0.00%	2.9926	0.00%	2.9926	0.00%
B2c	2.9921	2.9921	0.00%	2.9921	0.00%	2.9921	0.00%	2.9921	0.00%
B2d	2.9915	2.9914	0.00%	2.9914	0.00%	2.9914	0.00%	2.9914	0.00%
B2e	2.9910	2.9909	0.00%	2.9909	0.00%	2.9909	0.00%	2.9909	0.00%
B2f	2.9927	2.9674	0.85%	2.9926	0.00%	2.9674	0.85%	2.9926	0.00%
B2g	2.9923	2.9602	1.07%	2.9923	0.00%	2.9602	1.07%	2.9923	0.00%
B2h	2.9921	2.9532	1.30%	2.9921	0.00%	2.9532	1.30%	2.9921	0.00%
B2i	2.9918	2.9467	1.51%	2.9917	0.00%	2.9467	1.51%	2.9917	0.00%
B2j	2.9916	2.9405	1.71%	2.9915	0.00%	2.9405	1.71%	2.9915	0.00%
B2k	2.9945	2.9794	0.50%	2.9794	0.50%	2.9943	0.01%	2.9943	0.01%
B2l	2.9939	2.9679	0.87%	2.9679	0.87%	2.9933	0.02%	2.9933	0.02%
B2m	2.9935	2.9563	1.24%	2.9563	1.24%	2.9923	0.04%	2.9923	0.04%
B2n	2.9932	2.9450	1.61%	2.9450	1.61%	2.9912	0.07%	2.9912	0.07%
B2o	2.9920	2.9343	1.93%	2.9343	1.93%	2.9899	0.07%	2.9899	0.07%
Avg			0.84%		0.41%		0.44%		0.02%
Min			0.00%		0.00%		0.00%		0.00%
Max			1.93%		1.93%		1.71%		0.07%

**Table 7:** Evaluating the approximations for some C-sets.

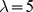		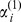		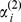		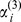		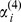	
Set	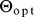	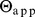	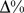	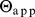	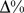	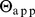	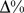	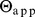	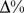
C1a	4.5810	4.5810	0.00%	4.5810	0.00%	4.5810	0.00%	4.5810	0.00%
C1b	4.4978	4.4717	0.58%	4.4717	0.58%	4.4717	0.58%	4.4717	0.58%
C1c	4.6002	4.4742	2.74%	4.4742	2.74%	4.5985	0.04%	4.5985	0.04%
C1d	4.5808	4.5806	0.00%	4.5806	0.00%	4.5806	0.00%	4.5806	0.00%
C1e	4.5826	4.5806	0.04%	4.5806	0.04%	4.5806	0.04%	4.5806	0.04%
C1f	4.5193	4.4531	1.46%	4.4531	1.46%	4.4531	1.46%	4.4531	1.46%
C1g	4.5964	4.4531	3.12%	4.5964	0.00%	4.4531	3.12%	4.5964	0.00%
C1h	4.5975	4.2370	7.84%	4.5913	0.13%	4.5102	1.90%	4.5973	0.00%
C1i	4.6003	4.5249	1.64%	4.5987	0.03%	4.5809	0.42%	4.6000	0.01%
Avg			1.94%		0.56%		0.84%		0.24%
Min			0.00%		0.00%		0.00%		0.00%
Max			7.84%		2.74%		3.12%		1.46%

## Conclusions and Final Remarks

In this paper, we examined the optimal routing problem in open finite acyclic queueing networks with a given general topology and multiple generally distributed servers. We determined the optimal routing probability vector that maximizes the throughput of an arbitrary configured network via a combination of the Generalized Expansion Method and Powell optimization tool. We presented numerical results showing the merits of the approach. Approximations for the routing probability vector are also presented and evaluated.

We have considered here only the throughput as the main performance measure. It would also be interesting to evaluate the behavior of the routing algorithm to minimize the cycle time, the work-in-process (WIP) or other performance measures. Topics for future research on the area include the routing in queueing networks with cycles, *e.g.*, to model many important industrial systems that have reverse streams of products due to re-work, or even the extension to 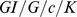 queueing networks.
